# Levels of anti-topoisomerase I antibody correlated with short onset of cardiopulmonary involvement in Thai systemic sclerosis patients

**DOI:** 10.1038/s41598-024-61159-3

**Published:** 2024-05-06

**Authors:** Kamonwan Mulalin, Ajanee Mahakkanukrauh, Siraphop Suwannaroj, Patnarin Pongkulkiat, Tippawan Onchan, Sawinee Kasa, Chingching Foocharoen

**Affiliations:** 1 Department of Medicine, Faculty of Medicine, Khon Kaen University, Khon Kaen, 40002 Thailand; 2 Clinical Laboratory Section, Faculty of Medicine, Khon Kaen University, Khon Kaen, 40002 Thailand

**Keywords:** Systemic sclerosis, Scleroderma and related disorders, Autoantibody, Anti-topoisomerase I, Interstitial lung disease, Autoimmunity, Rheumatology

## Abstract

Anti-topoisomerase-I antibody (ATA) is associated with disease severity and internal organ involvement in patients with systemic sclerosis (SSc). The correlation between ATA levels and the clinical course of SSc is unclear. We aimed to determine the correlation between ATA level and survival time and the onset of internal organ fibrosis in SSc patients. This historical cohort study was conducted in adult SSc patients with quantitative tests of ATA between January 2019 and December 2022. Patients with overlap syndrome and no quantitative ATA test were excluded. According to the sample size calculation, and 10% compensated for missing data, a total of 153 patients were needed. The respective mean age on the study date and median ATA level was 59.9 ± 11.3 years and 370 U/mL (range 195–652). Most cases (107 cases; 69.9%) were the diffuse cutaneous SSc subset. According to a multivariable analysis, the ATA titer had a negative correlation with the onset of cardiac involvement (Rho − 0.47, p = 0.01), and had a positive correlation with skin thickness progression (Rho 0.39, p = 0.04). Eleven cases exhibited ATA levels < 7 U/mL and outlier ATA levels were excluded, 142 cases were included in the sensitivity analysis, and multivariable analysis showed the correlation between early onset of ILD and cardiac involvement (Rho − 0.43, p = 0.03 and Rho − 0.51, p = 0.01, respectively). The ATA level was correlated with neither the survival time nor the onset of renal crisis in both analyses. High ATA levels were correlated with a short onset of ILD and cardiac involvement and the presence of extensive skin tightness. Quantitative tests of ATA could serve as an effective tool for identifying patients at risk of an unfavorable prognosis.

## Introduction

Systemic sclerosis (SSc) is a systemic disease in which fibroblasts predominate and produce excessive fibrosis of the skin and the internal organs^1^. The prevalence of systemic sclerosis in Asia is around 38–120 patients per million^2^. SSc is categorized into 2 subsets^3^—the limited cutaneous SSc (lcSSc) subset, in which skin thickness involves the face, distal to elbows, and below the knees, and the diffuse cutaneous SSc (dcSSc) subset, which involves the skin of trunk and that proximal to the elbows and knees. Abnormal fibrosis can be identified in internal organs (i.e., the heart, lungs, and gastrointestinal tract) and internal organ fibrosis is associated with a poor outcome. The ten-year survival rate in SSc is around 66%^2^; while the causes of death were mainly due to interstitial lung disease (ILD) and pulmonary hypertension (PHT).

Multiple autoantibodies have been reported in SSc, revealing associations between these antibodies and disease severity. Anti-topoisomerase-I antibody (ATA) is a well-known specific autoantibody in SSc. The prevalence was reported around 40–64% in dcSSc and 10–34% in lcSSc^4,5^. ATA is now included in the EULAR 2013 classification criteria for systemic sclerosis^6^. The antibody is also associated with a high risk of ILD and cardiac involvement, and some studies indicate that longstanding SSc with ATA positivity may precede certain cancers, such as lung cancer^7^. The quantitative level of ATA has been set so that the cut-off for classification of SSc is 20 U/mL^8^. The variations in the ATA level [determined by ELISA] with the extent of disease involvement have been reviewed. Sato et al. found variations in ATA level with extent disease involvement and seronegative conversion with disease remission, the level of ATA might relate to the severity of disease^9^. Kuwana et al. found that patients who lost ATA during follow-up had a better prognosis than those with persistent antibodies. However, no correlation has been reported between the level of the antibody and the severity of the disease^10^. ATA seroconversion was also revealed among Thais with SSc, albeit in a low prevalent and without any notable clinical associtaion^11^. As in heterogeneity of the previous studies, the ATA level and clinical course of SSc correlation is unclear, and there is no data to support whether the level of ATA has clinical relevance.

In Thailand, the prevalence of SSc was reported around 24 per 100,000 in 2017 and the incidence were 7.2, 7.6, 7.8 per 100,000 person-years in 2018, 2019, and 2020, respectively^12^. The majority of SSc were the dcSSc subset, which was reported at around 70%^13^ while the prevalence of ATA positive among SSc patients was around 70–87%^13,14^, which was higher than Caucasian and some Asian populations^15–24^. We hypothesized that a high ATA level would be correlated with poor outcomes in terms of early onset of internal organ fibrosis development and short survival time among SSc patients. If a high ATA is correlated with poor outcomes, then a quantitative test for ATA should be applied in daily practice instead of the traditional test which is usually immunoblotting or immunodiffusion in order to identify patients at risk of a poor prognosis.

## Methods

We did a historical cohort study among adult SSc patients ≥ 18 years old with follow-up at Srinagarind Hospital, Khon Kaen University, Khon Kaen, Thailand, between January 2019 and December 2022. The patients with the following conditions were excluded: (a) having an overlap with another connective tissue disease; and/or (b) no quantitative ATA test.

The primary outcome was determining the correlation between the ATA level and survival time in SSc patients. The secondary outcome was to determine the correlation between the ATA level and the onset of internal organ involvement in SSc patients.

### Data collection

**Figure 1:**
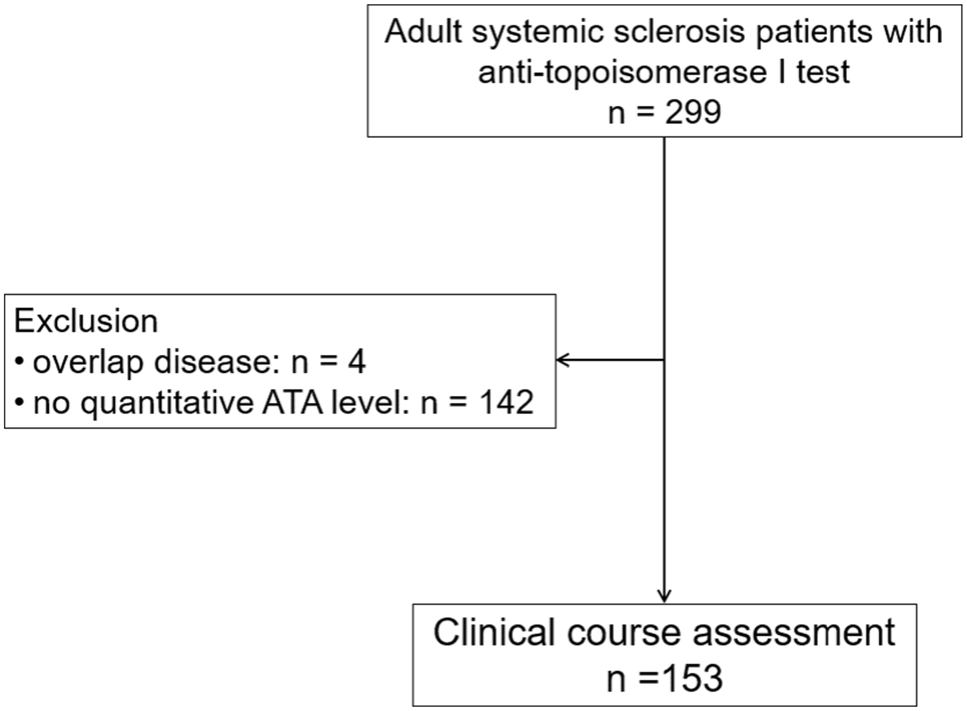
Study flow.

The demographic data were reviewed from the medical record, including age, sex, date of disease onset, clinical characteristics of SSc, disease duration at onset of internal organ involvement (ILD, PHT, renal crisis, cardiac involvement), routine laboratory tests including echocardiography, chest radiography, high resolute computed tomography (HRCT) of the lungs and lung function test, medical treatment, patient current status, and cause(s) of death if the patient died. (Fig. 1).

#### Laboratory method

The ATA level was measured by fluorescence enzyme immunoassay (FEIA), which was investigated by EliA Scl-70 (Phadia 250). The EliA Scl-70*S* wells were coated with human recombinant Scl-70 protein. The specimen was serum or plasma (EDTA). Samples had to be diluted with EliA sample diluent at a ratio of 1:100. If ATA was present in the patient's specimen, antibodies to Scl-70 were bound to the specific antigen. After washing away non-bound antibodies, enzyme-labeled antibodies against human IgG antibodies (EliA IgG Conjugate) were added to form an antibody-conjugate complex. After incubation, the non-bound conjugate was washed away, and the bound complex was incubated with a development solution. After stopping the reaction, the fluorescence in the reaction mixture was measured. The assay directly measured the amount of antibody of interest bound to the antigen coating the EliA well; thus, the higher the fluorescent signal value detected, the higher the amount of antibody bound and detected in the sample tested^25^.

#### Operational definitions

Skin assessment was conducted using modified Rodnan skin score (mRSS). The mRSS assessments were performed by (a) expert rheumatologists who validated the assessment^26^, and (b) physicians, internists, or fellows in rheumatology who had received training from expert rheumatologists or were supervised by them. The intra- and inter-observer variability for mRSS assessment in our center has been previously validated^26^. Additionally, we have implemented a skin model for mRSS assessment to ensure quality control for physician who are not expert rheumatologists^27^.

A digital ulcer was defined when there was a painful denuded area with well-demarcated borders located on the volar aspect of the fingers^28^. Hand deformity was defined when the finger joints had flexion contractures resembling claw deformities^29^. The definition of ILD was fulfilled when either interstitial fibrosis was detected by HRCT or chest X-ray^30^. Pulmonary arterial hypertension (PAH) was diagnosed when either the mean pulmonary arterial pressure (mPAP) was > 20 mmHg at rest with a pulmonary artery wedge pressure of ≤ 15 mmHg with a pulmonary vascular resistance of > 2 Wood units, as confirmed by right heart catheterization^31^, or RVSP > 50 mmHg^32^, TRVmax > 3.4 m/s by echocardiogram^33^. PHT due to ILD (PH-ILD) was defined by mPAP between 20 and 34.9 mmHg, and a forced vital capacity (FVC) < 70% or forced expiratory volume 1 (FEV1) < 60% predicted or ≥ 20% involvement of ILD evaluated by HRCT. Esophageal involvement was defined when any esophageal symptoms of SSc presented (i.e., esophageal dysphagia, heartburn, or reflux symptoms). Stomach involvement was defined by the symptom of early satiety or vomiting^34^. Intestinal involvement was determined by symptoms of diarrhea, bloating, malabsorption, constipation, and/or ileus or pseudo-intestinal obstruction. Cardiac involvement was defined if the patient had any of the following: (a) myocardial involvement; defined when the left ventricular ejection fraction ≤ 50%; (b) pericardial effusion; detected by echocardiogram or CT scan; (c) heart failure; recorded by physician; and (d) cardiac arrhythmia. Renal crisis was indicated when there was (a) a rapid, progressive, rise in serum creatinine; (b) the abrupt onset of hypertension; and/or, (c) microangiopathic hemolytic anemia.

The date of SSc onset was the date of the first non-Raynaud symptoms of SSc. The date of onset of internal organ involvement was the date of first identification of having internal organ involvement as defined above. The end-date referred to the end-date of the study (December 31, 2022), or alternatively, the date at the last follow-up. The duration of disease was calculated by subtracting of the end date from the date of SSc onset. The duration of disease at onset of internal organ involvement was the interval between the date of SSc onset and the date of internal organ involvement. The health status of patients lost to follow-up were retrieved from The Bureau of Registration Administration, and the information was reviewed, and the cause of death ascertained by a physician. Time-to-event (death) was the time calculated by subtracting the date of death from the date of SSc onset.

### Statistical methods

Sample size was calculated by using a correlation coefficient of 0.237 between disease activity and line immunoblot assay data according to the recent literature^8^. With 80% power at a significance level of 0.05, the sample size was determined to be 137 cases. To compensate for potentially 10% missing data or incomplete data, we recruited 153 patients into the study.

Clinical data were divided into dichotomous, polytomous, or continuous variables. The correlation between the ATA level and survival time was evaluated using a Pearson correlation or Spearman Rank correlation, as appropriate. The correlation between the ATA level and duration of disease at onset of internal organ involvement was evaluated using the same method. A quantitative ATA test was performed using the FEIA technique with a cut-off of < 7 U/mL being considered a negative test, 7–10 U/mL being equivocal, and > 10 U/mL being positive^25^. With respect to our study methodology, we included all detected antibody levels to analyze their correlation with clinical outcomes during the ATA test and at the last follow-up. Additionally, a sensitivity analysis was conducted, excluding ATA levels < 7 U/mL classified as negative for ATA and outlier ATA level, using the same method. p-values < 0.05 were considered statistically significant. The data were analyzed using STATA version 16.0 (StataCorp., College Station, TX, USA).

### Ethics approval and consent to participate

The Human Research Ethics Committee of Khon Kaen University approved the study as per the Helsinki Declaration and the Good Clinical Practice Guidelines (HE651508). The Human Research Ethics Committee of Khon Kaen University waived the requirement for informed consent because of the retrospective nature of the study. Participant privacy was protected by anonymized data and kept confidentiality. The study methods were performed in accordance with the Helsinki Declaration statement.

## Results

A total of 153 patients who were tested for ATA antibody levels were included in the study. The respective mean age at onset and age at the first visit was 54.6 ± 13 and 59.9 ± 11.3 years. The median duration of disease was 2.2 (IQR 1.3–6.0) years. Most of the patients were female (99 cases; 64.7%) and had dcSSc (107 cases; 69.9%). The median ATA level was 370 (IQR 195–652) U/mL. Among those tested for ATA antibody levels, 11 cases exhibited ATA levels < 7 U/mL and were considered outliers, thus they were excluded. Therefore, 142 cases were included in the sensitivity analysis. The demographic data and clinical characteristics of SSc on the date of the ATA test for all those tested for ATA antibody levels and those who were outlier of ATA levels are presented in Table 1.

**Table 1:** Baseline characteristics.

Characteristics	All patients (N = 153)	Excluding who had ATA < 7 U/mL and outlier (N = 142)
Age at onset, years, mean (SD)	54.6 (13.0)	55.0 (13.0)
Disease duration, years, median (IQR)	2.2 (1.3–6.0)	2.2 (1.3–6.0)
Female, N (%)	99 (64.7)	92 (64.8)
BMI, kg/m^2^, mean (SD)	21.6 (3.6)	21.8 (3.7)
Diffuse cutaneous SSc, N (%)	107 (69.9)	103 (72.5)
Comorbidities, N (%)	110 of 153 (72.4)	102 of 141 (72.3)
Diabetes mellitus	30 (27.3)	29 (28.4)
Hypertension	28 (25.5)	27 (26.5)
Dyslipidemia	41 (37.3)	38 (37.3)
CKD	14 (12.7)	13 (12.8)
Hypothyroid	9 (8.3)	9 (8.9)
Cancer	4 (3.6)	4 (3.9)
Osteoporosis	6 (5.5)	6 (5.9)
Vitamin D deficiency	15 (13.6)	12 (11.8)
Other	61 (55.5)	57 (55.9)
ATA level, U/mL, median (IQR)	370 (195–652)	376.5 (240–675)
Clinical on the date of ATA test		
WHO functional class, N (%)	126 (100)	118 (100)
I	55 (43.7)	50 (42.4)
II	60 (47.6)	58 (49.2)
III	7 (5.6)	6 (5.1)
IV	4 (3.2)	4 (3.4)
SSc clinical characteristics, N (%)		
Raynaud’s phenomenon	102 of 138 (73.9)	92 of 128 (71.9)
Digital ulcer	41 of 127 (32.3)	37 of 119 (31.1)
Digital gangrene	3 of 114 (2.6)	3 of 107 (2.8)
Telangiectasia	36 of 119 (30.3)	33 of 111 (29.7)
Calcinosis cutis	5 of 120 (4.2)	5 of 113 (4.4)
Salt and pepper skin appearance	84 of 125 (67.2)	81 of 118 (68.6)
Edematous skin	64 of 121 (52.9)	60 of 113 (53.1)
Skin tightness	118 of 142 (83.1)	108 of 131 (82.4)
Tendon friction rub	25 of 118 (21.2)	25 of 111 (22.5)
Hand deformity	33 of 112 (29.5)	31 of 105 (29.5)
Arthritis	30 of 133 (22.6)	28 of 125 (22.4)
Muscle weakness	9 of 119 (7.6)	8 of 111 (7.2)
Esophageal involvement	73 of 153 (47.7)	70 of 142 (49.3)
Stomach involvement	19 of 115 (16.5)	18 of 108 (16.7)
Intestinal involvement	25 of 153 (16.3)	25 of 142 (17.6)
Weight loss	65 of 123 (52.9)	59 of 114 (51.8)
mRSS, mean (SD)	9.8 (8.1)	9.9 (8.2)
Laboratory		
Hemoglobin, g/dL, mean (SD)	11.7 (1.7)	11.7 (1.7)
Creatinine, mg/dL, mean (SD)	0.9 (0.4)	0.9 (0.4)
Albumin, g/dL, mean (SD)	3.8 (0.6)	3.8 (0.6)
Creatinine kinase, U/L, median (IQR)	140.5 (84.5–264.5)	141 (81.5–266.5)
CRP, mg/L, median (IQR)	6.9 (2.3–14.7)	7.1 (2.3–15.3)
SR, mm/h, mean (SD)	65.9 (31.4)	66.7 (30.9)
hsTroponin T, ng/L, median (IQR)	38.9 (11.9–169.0)	46.4 (12.2–232.0)
FVC, %, mean (SD)	70.4 (14.8)	70.1 (14.7)
6MWT, metre, mean (SD)	430.0 (95.9)	429.4 (98.2)
EF, %, mean (SD)	69.5 (10.0)	69.7 (10.1)
RVSP, mmHg, mean (SD)	34.6 (12.0)	34.3 (11.9)
Clinical at the last follow up		
BMI, kg/m^2^, median (IQR)	20.8 (18.5–23.0)	21.0 (18.5–23.0)
Functional class, N (%)		109 (100)
I	51 (44.0)	46 (42.2)
II	48 (41.4)	46 (42.2)
III	11 (9.5)	11 (10.1)
V	6 (5.2)	6 (5.5)
SSc clinical characteristics, N (%)		
Raynaud phenomenon	69 of 119 (58.0)	63 of 111 (56.8)
Digital ulcer	26 of 116 (22.4)	25 of 108 (23.2)
Digital gangrene	1 of 115 (0.9)	1 of 107 (0.9)
Telangiectasia	53 of 113 (46.9)	49 of 106 (46.2)
Calcinosis cutis	8 of 114 (7.0)	8 of 107 (7.5)
Salt and pepper skin appearance	71 of 114 (62.3)	68 of 107 (63.6)
Edematous skin	22 of 113 (19.5)	20 of 106 (18.9)
Skin tightness	89 of 122 (73.0)	84 of 114 (73.7)
Tendon friction rub	18 of 114 (15.8)	18 of 107 (16.8)
Hand deformity	56 of 114 (49.1)	52 of 107 (48.6)
Arthritis	14 of 116 (12.1)	14 of 108 (13.0)
Muscle weakness	5 of 114 (4.4)	5 of 107 (4.7)
Esophageal involvement	48 of 153 (31.4)	45 of 142 (31.7)
Stomach involvement	13 of 115 (11.3)	12 of 107 (11.2)
Intestinal involvement	21 of 153 (13.7)	18 of 142 (12.7)
Weight loss	28 of 115 (24.4)	27 of 107 (25.2)
mRSS, mean (SD)	9.8 (9.4)	10.1 (9.5)
Laboratory		
Hemoglobin, g/dL, mean (SD)	11.9 (1.7)	11.9 (1.7)
Creatinine, mg/dL, mean (SD)	1.02 (1.0)	1.035 (1.033)
Albumin, g/dL, mean (SD)	3.9 (0.5)	3.9 (0.5)
Creatinine kinase, U/L, median (IQR)	97.0 (60.0–180.0)	97.0 (60.0–179.0)
CRP, mg/L, median (IQR)	3.4 (1.2–9.6)	3.5 (1.2–9.6)
ESR, mm/h, mean (SD)	64.9 (30.8)	65.3 (31.5)
hsTroponin T, ng/L, median (IQR)	21.5 (11.9–130)	24.1 (12.0–153.0)
FVC, %, mean (SD)	69.7 (15.4)	69.6 (15.2)
6MWT, metre, mean (SD)	432.6 (92.6)	432.2 (94.5)
EF, %, mean (SD)	67.2 (13.7)	66.8 (14.0)
RVSP, mmHg, mean (SD)	34.7 (10.6)	33.1 (9.6)

*SD* standard deviation, *N* number, *SSc* systemic sclerosis, *CKD* chronic kidney disease, *ATA* anti topoisomerase-I antibody, *BMI* body mass index, *CRP* C-reactive protein, *ESR* erythrocyte sedimentation rate, *hsTnT* high sensitivity troponin T, *FVC* forced vital capacity, *6MWT* Six Minute Walk Test, *EF* ejection fraction, *RVSP* right ventricular systolic pressure, *mRSS* modified Rodnan skin score, *IQR* interquartile range.

### Primary outcome

Thirty-five of the patients did not survive (22.9%). Non-SSc related death was the most common cause of death (21 cases; 60.0%), of which pneumonia was the most common cause (28.6%), while unspecified organ involvement was the most common cause of SSc-related death (7 cases). The causes of death are presented in Table 2.

**Table 2:** Outcomes of the patient.

Data	All patients (N = 153)	Excluding who had ATA < 7 U/mL and outlier (N = 142)
Events		
ILD, N (%)	124 (81.1)	115 (81.0)
Duration of disease at onset of ILD, years, median (IQR)	0.7 (0.3–2.7)	0.7 (0.3–2.7)
Cardiac involvement	45 (29.4)	42 (29.6)
Pericardial effusion	39 (25.5)	37 (26.1)
Heart failure	5 (3.3)	4 (2.8)
Arrhythmia	2 (1.3)	2 (1.4)
Duration of disease at onset of cardiac involvement, years, median (IQR)	0.8 (0.3–1.8)	0.8 (0.3–1.7)
Renal crisis, N (%)	13 (8.5)	13 (9.2)
Duration of disease at onset of renal crisis, years, median (IQR)	0.6 (0.3–0.9)	0.6 (0.3–0.9)
PHT, N (%)	12 (7.8)	11 (7.8)
Duration of disease at onset of PHT, years, median (IQR)	1.1 (0.9–1.8)	1.1 (0.8–1.6)
Current status N (%)		
Alive	118 (77.1)	107 (75.4)
Non-survive	35 (22.9)	34 (23.9)
Loss follow up	1 (0.7)	1 (0.7)
Causes of death		
SSc related	14 (40)	13 (38.2)
SSc non-related	21 (60)	21 (61.8)
SSc related death		
Unspecified organ involvement	7 (20.6)	6 (17.6)
Cardiac involvement	3 (8.6)	3 (8.8)
Renal crisis	3 (8.6)	3 (8.8)
ILD	1 (2.9)	1 (2.9)
SSc non-related		
Cancer	2 (5.7)	2 (5.9)
Pneumonia	10 (28.6)	10 (29.4)
Sepsis	2 (5.7)	2 (5.9)
CKD	3 (8.6)	3 (8.8)
Other	4 (11.4)	4 (11.8)
Duration of disease on date of death, years, median (IQR)	1.1 (0.5–2.6)	1.2 (0.5–2.6)

*SD* standard deviation, *N* number, *CKD* chronic kidney disease, *ILD* interstitial lung disease, *PHT* pulmonary hypertension, *SD* standard deviation, *IQR* interquartile range.

Mean disease duration on the death date was 1.1 years (IQR 0.5–2.6). There was no correlation between the ATA level and survival time with a Rho of − 0.22 (p = 0.22) (Table 3).

**Table 3:** Correlation between ATA level and clinical parameters.

Clinical parameters	All patients (N = 153)		Excluding who had ATA < 7 U/mL and outlier (N = 142)	
	Rho	p-value	Rho	p-value
Survival time	− 0.22	0.22	− 0.33	0.06
Disease duration at onset of ILD	− 0.21	0.02*	− 0.19	0.04*
Disease duration at onset of PHT	− 0.48	0.11	− 0.74	0.01*
Disease duration at onset of renal crisis	− 0.07	0.83	− 0.07	0.83
Disease duration at onset of cardiac involvement	− 0.37	0.01*	− 0.50	< 0.001*
Age at onset	0.06	0.48	0.43	0.62
Age at death	0.16	0.35	0.16	0.37
mRSS on the date of ATA test	0.35	< 0.001*	0.36	< 0.001*
mRSS on last follow up	0.30	< 0.001*	0.29	0.002*

*Statistical significant.

*ILD* interstitial lung disease, *PHT* pulmonary hypertension, *mRSS* modified Rodnan skin score.

### Secondary outcome

ILD was the most common internal organ involvement during follow-up (124 cases; 81.1%), followed by cardiac involvement (45 cases; 29.4%), renal crisis (13 cases; 8.5%), and PHT (12 cases; 7.8%). The duration of disease at onset of ILD, PHT, renal crisis, and cardiac involvement are presented in Table 2.

By univariate analysis, the ATA level correlated with early onset of ILD and cardiac involvement with a Rho of − 0.21 (p = 0.02) and − 0.37 (p = 0.01), respectively. We did not find any correlation between the ATA level and disease duration at onset of PHT and renal crisis (Table 3). We also found a significant positive correlation between the ATA level and mRSS (Rho 0.30), salt and pepper skin (Rho 0.26), and muscle weakness (Rho 0.21), but a negative correlation with intestinal involvement (Rho − 0.19) and serum albumin (Rho − 0.18) (Supplementary Table 1).

By multivariable analysis (adjusted with disease duration at onset of ILD, disease duration at onset of cardiac involvement, mRSS on the date of ATA test, mRSS on last follow up), the ATA levels correlated with early onset of cardiac involvement (Rho − 0.47, p = 0.01) and mRSS at last follow up (Rho 0.39, p = 0.04). By excluding outlier and who had ATA level < 7 U/mL, multivariable analysis showed correlation between early onset of ILD and cardiac involvement (Rho − 0.43, p = 0.03 and Rho − 0.51, p = 0.01, respectively). The multivariable analysis of the correlation between ATA level and clinical parameters was presented in Table 4.

**Table 4:** Multivariable analysis of the correlation between ATA level and clinical parameters.

Clinical parameters	All patients		Excluding who had ATA < 7 U/mL and outlier	
	Rho	p-value	Rho	p-value
Disease duration at onset of ILD	− 0.36	0.06	− 0.43	0.03*
Disease duration at onset of cardiac involvement	− 0.47	0.01*	− 0.51	0.01*
mRSS on the date of ATA test	0.20	0.32	0.19	0.37
mRSS on last follow up	0.39	0.04*	0.35	0.08

*Statistical significant.

*ILD* interstitial lung disease, *mRSS* modified Rodnan skin score.

## Discussion

This study demonstrated the correlation between ATA levels and disease outcomes among Thai SSc patients. ATA is a well-known specific autoantibody in SSc, and the presence of ATA is not only helpful for early diagnosis of SSc, but also predictive of disease outcome. The significance of the quantitative ATA level has been tested in our SSc patients since 2019; however, it has not replaced the conventional test that yields positive or negative results. We collected clinical outcomes during follow-up to determine the correlations. During follow-up, some clinical features observed during the last visit may differ from those noted during the visit when ATA was tested. Such variances were attributed to the subsiding of clinical features following treatment during the follow-up period. Therefore, clinical symptoms potentially reversible after treatment were not cumulative.

The most common organ involvements among our events of interest were ILD (81%), followed by cardiac involvement (29%), death (23%), renal crisis (9%), and PHT (8%). During a median follow-up time of 2.2 years, the shortest to longest disease duration at the time of development of the events of interest was renal crisis (0.6 years), ILD (0.7 years), cardiac involvement (0.8 years), death (1.1 years), and PHT (1.1 years). The finding was compatible with the causes of death due to SSc-related death in which renal crisis and cardiac involvement were the common causes of SSc-related death. Although ILD was the most common major internal organ involvement among our SSc patients, the prognosis in this study seemed to be better than among those who had cardiac involvement and renal crisis. This might be influenced by the collection of lung fibrosis and ILD severity across all levels.

The ATA level could serve as a biomarker for predicting the onset of ILD and cardiac involvement in SSc. In our sensitivity analysis in those who had cut-off ATA levels ≥ 7 U/mL and outlier ATA levels, we observed a negative correlation between the ATA levels and disease duration at the onset of ILD and cardiac involvement. In addition, the ATA levels also tended to be negatively correlated with disease at the onset of ILD among all tested ATA levels. The pathogenesis of ILD involves excessive fibroblast activation and overproduction of extracellular matrix, finally leading to pulmonary fibrosis^35^. ATA is known as a major antibody among Thais, particularly among patients with the dcSSc subset in which fibrosis predominates^13,14,36^. According to the pathogenesis, ATA can also trigger genes which produce collagen expression, so the fibrosis is worsen. Moreover, this antibody can produce interferon alpha via CD8+ activation, potentially leading to lung tissue damage in SSc patients^4^ and might trigger inflammation in pathogenesis of SSc-ILD^37^. Hence, it should not be a surprise that the higher the level of ATA correlated to a shorter duration of onset of ILD. Fibrosis was also discovered in the myocardium. Nogradi et al. found that right atrium (RA) stiffness is associated with mortality in SSc patients, even in the absence of pulmonary hypertension^38^. This might explain our result regarding ATA levels and the short duration to cardiac involvement. Unfortunately, we did not collect data on RA stiffness. Based on our findings, ATA levels can offer a more comprehensive understanding of the correlation between the onset of ILD and cardiac involvement. We suggest conducting an antibody test using quantitative ATA level testing, if feasible, rather than a qualitative test that solely provides a positive or negative of the antibody.

We identified significant positive correlation between the ATA levels and skin thickness progression during follow-up, as assessed by mRSS, irrespective of the cut-off ATA levels. In the sensitivity analysis, ATA levels appeared to be correlated with mRSS during follow-up in those who had cut-off ≥ 7 U/mL and were not considered outliers. Our findings were similar to a study by Hu et al.^39^. The authors reported an association between the ATA IgG titer levels and mRSS in 59 SSc patients and also revealed that of three patients with high ATA titers and inactive disease, two of them subsequently exhibited higher mRSS after 7–11 months follow-up^39^. Nevertheless, a study by Hu et al. and ours did not investigate the longitudinal change of ATA levels and the correlation with mRSS. We cannot therefore answer whether increasing ATA levels by longitudinal follow-up correlate with an increasing mRSS. Further longitudinal follow-up of ATA levels is suggested. The correlation between ATA levels and skin thickness progression might be explained by the pathogenic nature of ATA antibody. Piantoni et al. demonstrated that ATA acts by binding with fibroblasts and inducing excessive fibroblast production^4^. However, the exact pathogenic process of ATA on the skin remains unknown.

Although ATA was reported to be a predictor of mortality in SSc^40,41^, we did not find any correlation between the ATA level and survival time. This finding might be related to the short duration of follow-up in our patients. Further, longer observations should be conducted. Our results show that the ATA level could be a good predictor for SSc monitoring in order to predict patients with a poor prognosis, so early detection and treatment should be done in order to decrease morbidity in SSc patients.

Our study had some limitations, including: (a) The follow-up duration has been short since ATA level became available in 2019, potentially limiting our ability to observe survival outcomes adequately, (b) the absence of disease activity and treatment effect evaluation, so we did not see the correlation between ATA levels and other parameters, (c) the data is incomplete due to the retrospective nature of the study and the diverse range of physicians assessing the patients, (d) a single-center study, limiting the generalizability of the findings, (e) due to facility limitations, there is no precise interval for the screening protocol. Additionally, a normal chest radiograph does not necessarily indicate the absence of ILD. However, our patients with suspected ILD symptoms and normal chest radiographs underwent evaluation for ILD using HRCT, and (f) left ventricular diastolic dysfunction or the presence of subclinical myocardial dysfunction, which is often detected by echocardiography without significant symptoms, were not included in our analysis. Therefore, we were unable to determine whether there was a correlation between ATA levels and the onset of those cardiac problems. The strengths of our study are (a) adequate sample size by sample size calculation, lending confidence that our findings were analyzed with sufficient data and information, and (b) the first study to determine the correlation between ATA levels and the onset of internal organ involvement and survival time. The quantitative ATA test might be used as a guide for assessing and monitoring disease complications and/or facilitating early intervention.

## Conclusion

ATA level was significantly correlated with clinical outcomes of SSc; in terms of early onset to ILD and cardiac involvement. Quantitative tests for ATA might be a good tool for identifying patients at risk of a poor prognosis. Longitudinal studies into changes in the ATA level and long-term clinical outcomes should be done.

### Supplementary Information

Supplementary Table 1.

Supplementary Table 2.

## Data Availability

All data generated or analysed during this study are included in this published article and its supplementary information files (Supplementary Table 2).
