# Supplementary material for: Levels of anti-topoisomerase I antibody correlated with short onset of cardiopulmonary involvement in Thai systemic sclerosis patients
Source: Sci Rep. 2024 May 6;14:10354. doi: 10.1038/s41598-024-61159-3 (PMC11074118; doi:10.1038/s41598-024-61159-3)
Supplement: Supplementary file 1 — Supplementary Table 1. [file 41598_2024_61159_MOESM1_ESM.docx]

**Supplementary Table 1**. Correlation between ATA level and clinical parameters

| **Clinical parameters** | **All patients**  **(N = 153)** | |
| --- | --- | --- |
|  | Rho | p-value |
| Clinical at last follow up | | |
| BMI | -0.03 | 0.75 |
| mRSS | 0.30 | <0.001* |
| Functional class | -0.03 | 0.76 |
| Raynaud phenomenon | 0.06 | 0.51 |
| Digital ulcer | 0.02 | 0.80 |
| Digital gangrene | -0.08 | 0.37 |
| Telangiectasia | 0.14 | 0.14 |
| Calcinosis cutis | 0.02 | 0.84 |
| Salt and pepper skin | 0.26 | 0.01* |
| Edematous skin | 0.12 | 0.21 |
| Tendon friction rub | 0.13 | 0.16 |
| Hand deformity | 0.11 | 0.26 |
| Arthritis | 0.12 | 0.19 |
| Muscle weakness | 0.21 | 0.02* |
| Esophageal involvement | -0.03 | 0.71 |
| Intestinal involvement | -0.19 | 0.02* |
| Stomach involvement | -0.01 | 0.94 |
| Weight loss | 0.31 | 0.74 |
| Hemoglobin | 0.03 | 0.78 |
| Serum albumin | -0.18 | 0.04* |
| Creatinine kinase | 0.10 | 0.30 |
| High sensitivity troponin T | 0.21 | 0.12 |
| NT-proBNP | 0.08 | 0.62 |

*statistical significant
BMI body mass index, mRSS modified Rodnan skin score, NT-proBNP N-terminal prohormone of brain natriuretic peptide
